# Nature’s Load-Bearing Design Principles and Their Application in Engineering: A Review

**DOI:** 10.3390/biomimetics9090545

**Published:** 2024-09-09

**Authors:** Firas Breish, Christian Hamm, Simone Andresen

**Affiliations:** Alfred-Wegener-Institut Helmholtz-Zentrum für Polar- und Meeresforschung, 27570 Bremerhaven, Germany; christian.hamm@awi.de (C.H.); simone.andresen@awi.de (S.A.)

**Keywords:** biomimetics, mechanics, load-bearing, hierarchical materials, functional gradients, cellular structures, form and function

## Abstract

Biological structures optimized through natural selection provide valuable insights for engineering load-bearing components. This paper reviews six key strategies evolved in nature for efficient mechanical load handling: hierarchically structured composites, cellular structures, functional gradients, hard shell–soft core architectures, form follows function, and robust geometric shapes. The paper also discusses recent research that applies these strategies to engineering design, demonstrating their effectiveness in advancing technical solutions. The challenges of translating nature’s designs into engineering applications are addressed, with a focus on how advancements in computational methods, particularly artificial intelligence, are accelerating this process. The need for further development in innovative material characterization techniques, efficient modeling approaches for heterogeneous media, multi-criteria structural optimization methods, and advanced manufacturing techniques capable of achieving enhanced control across multiple scales is underscored. By highlighting nature’s holistic approach to designing functional components, this paper advocates for adopting a similarly comprehensive methodology in engineering practices to shape the next generation of load-bearing technical components.

## 1. Introduction

Striving to bear the weight of a grape cluster at peak ripeness (a trivial task for us, but a Herculean effort for a delicate grapevine on the Lebanese mountainside every end of September), braving relentless, howling wind gusts (like a kite in a hurricane, if the kite were a proud oak tree in the North German city of Bremerhaven in November), enduring a sudden, mighty club hit from a mantis shrimp at the shallow coral reefs off the coast of Okinawa, Japan, at any time of the year (imagine a tiny boxer with a punch that could break glass), leaping over a narrow creek in a single bound to escape a hungry pack of wild dogs in the African savannah (an antelope performing an Olympic long jump, with the added pressure that the sandpit is a matter of life and death). These scenarios are not just snippets from a nature documentary’s most dramatic moments; they are regular occurrences in the natural world. And they all involve substantial mechanical loads acting on biological structures.

Looking at nature to develop lightweight load-bearing engineering components is highly compelling due to the following key attributes of natural structures:

- Optimized through natural selection and tested diversity: Nature has been shaped by countless iterations of evolution, resulting in structures finely tuned to withstand the expected mechanical loads throughout an organism’s lifetime [1]. With several million organisms, nature offers a diverse array of optimized solutions under real conditions. Biological structures, though they might differ significantly from technical products, present coherent solutions pre-optimized for specific applications. By studying these evolutionary outcomes, engineers can leverage nature’s proven solutions to create structures that are equally well tailored to their intended operational mechanical load cases [2].
- Sustainable and resource-efficient: Nature excels in sustainable and resource-efficient solutions. Organisms evolve to minimize waste, maximize energy efficiency, and operate in harmony with their ecosystems. The combination of minimal material input and the use of materials that can be produced and degraded under physiological conditions, such as cellulose, chitin, lignin, silicate, calcium phosphate, and calcium carbonate, is particularly attractive for developing products within a circular economy framework [3]. These principles offer solutions for lightweight engineering designs that reduce ecological impact and promote sustainability—crucial in an era of resource scarcity and environmental degradation.
- Multi-functional, adaptable, and robust: Nature’s designs often display multi-functionality and adaptability, enabling efficient performance in diverse environments. For instance, diatom shells exhibit low weight, high mechanical resilience, and high permeability [4]. Such integrated functions have been challenging to optimize using conventional calculation tools. Technical lightweight products often assume very specific load cases, and methods such as topology optimization can provide excellent solutions for these scenarios. However, in reality, loads are versatile and include unexpected situations [5]. Natural lightweight structures are typically adapted to such variability by displaying complex, interconnected designs with minimal weaknesses, making them robust against deviations from anticipated load cases. This adaptability and robustness is especially relevant in load-bearing engineering components, which often need to respond to multiple loads and adapt to changing conditions.

The concept of drawing inspiration from nature for structural design dates back to ancient civilizations. However, it was not until the 20th century that researchers, like Julian Vincent, directed their works towards studying the mechanical performance of different biological materials and structures [6,7,8,9]. Their research effectively bridged the gap between biology and structural engineering, ultimately fostering the rapid growth in the field in the first decade of the 21st century [10].

**Figure 1:**
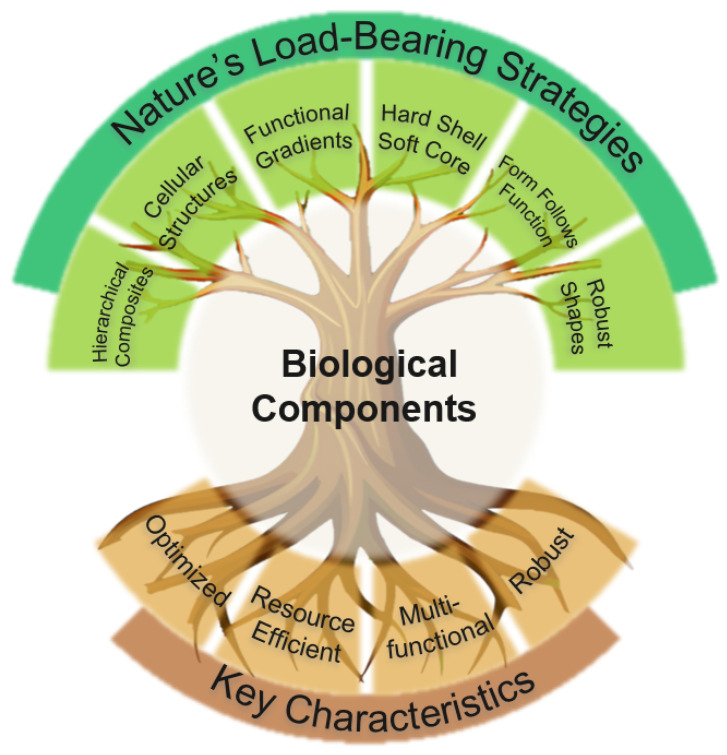
Illustration of nature’s load-bearing strategies as categorized in this study, highlighting the interconnectedness of these strategies within biological components to achieve their beneficial characteristics.

This paper provides an overview of key principles, strategies, and features that have evolved in nature for efficient mechanical load handling, as well as successful applications of these strategies in the technical domain. These strategies are organized into six main categories: hierarchically structured composites, cellular structures, functional gradients, hard shell–soft core architecture, form follows function, and robust geometric shapes. This categorization is merely intended to provide structure; however, in nature, these strategies typically occur in combination rather than in isolation. Nature’s load-bearing strategies, as categorized in our study, and their interconnectedness within biological components to achieve their beneficial characteristics, are illustrated in Figure 1.

One overarching aim is to present these strategies in a way that highlights nature’s holistic approach to ‘designing’ functional components from the nano- to the macro-scale and to encourage the adoption of a similar philosophy in the design of engineering load-bearing components.

## 2. Hierarchically Structured Composites

The trade-off between a material’s strength and toughness presents a significant challenge for researchers and engineers. Increasing a material’s strength often involves restricting dislocation movement, which can reduce ductility as the material becomes less capable of undergoing plastic deformation without fracturing, thus compromising overall toughness [11]. Nature, however, elegantly overcomes this strength–ductility trade-off in fibrous bio-polymers like collagen and viscid spider silk through sophisticated hierarchical structures, ranging from molecular arrangements to macroscopic fibers [12,13]. Under tension, these bio-polymers display a J-shaped stress–strain curve, where molecular uncoiling and unkinking lead to considerable deformation under low stress. At higher stresses, the polymer chains unfurl, straighten, and stretch as they slide past each other, resulting in a stiffening effect [14]. This combination of low initial energy expenditure and stiffening near the breaking point is key to spider silk’s remarkable toughness [15,16,17]. It also results in high damping capacity across varying deformation rates, enabling the secure capture of high-velocity prey without rebound [18].

Furthermore, Cranford et al. (2012) [19] demonstrated that the nonlinear mechanical response of spider silk, characterized by initial softening followed by strain-induced stiffening, is crucial for localizing damage and enhancing the robustness of spider webs. The softening phase helps distribute stress and absorb energy, preventing immediate failure under moderate loads. As the load increases, the silk stiffens, concentrating deformation on the affected thread, thereby confining damage and preserving the web’s overall structural integrity. This serves as an excellent example of how material properties in natural systems dictate structural behavior, and how natural materials and geometries work in unison to enhance the robustness of the system as a whole.

Other natural materials that exhibit strain stiffening include biological tissues like skin, tendons, and the extracellular matrix [20,21,22]. These tissues remain soft and flexible under low strains but rapidly stiffen during large deformations to prevent damage [23].

Researchers have highlighted the potential of mimicking spider silk to develop synthetic fibers for technical applications [24,25,26,27]. Recent advancements in production methods have led to the successful development of synthetic fibers with comparable properties [17,28,29,30,31,32]. In a recent study, researchers replicated the stress response mechanisms of spider silk to create a high-strength, ultra-tough film that outperformed other bio-based materials and even conventional plastics, making it a promising candidate for replacing plastics in efforts to reduce environmental waste [32].

Additionally, significant efforts have been made in developing biomimetic smart materials with strain-stiffening properties and other self-adaptive mechanisms in response to mechanical loading [33,34,35,36]. In one study, researchers developed biomimetic elastomers inspired by the strain-stiffening behavior of biological tissues, resulting in ultra-stretchable and tough materials. These materials show great potential for applications in wearable device technology [36].

Nature also addresses the strength–ductility trade-off through the structural biological materials found in mollusk shells, diatoms, sea sponges, teeth, tusks, bone, antlers, crab exoskeletons, and insect cuticles, all of which share a common composition. These materials combine bio-polymers (like collagen, keratin, elastin, cellulose, and chitin), which contribute to toughness and resilience, and mineral phases (such as calcium carbonate, carbonated hydroxyapatite, or silica), which enhance the material’s hardness and stiffness [37,38,39]. Biological composite materials have independently evolved in various other instances, including chitin and chitosan-based composites found in arthropod exoskeletons, fish scales, fungal cell walls, certain algae species, and select marine sponge skeletons.

**Figure 2:**
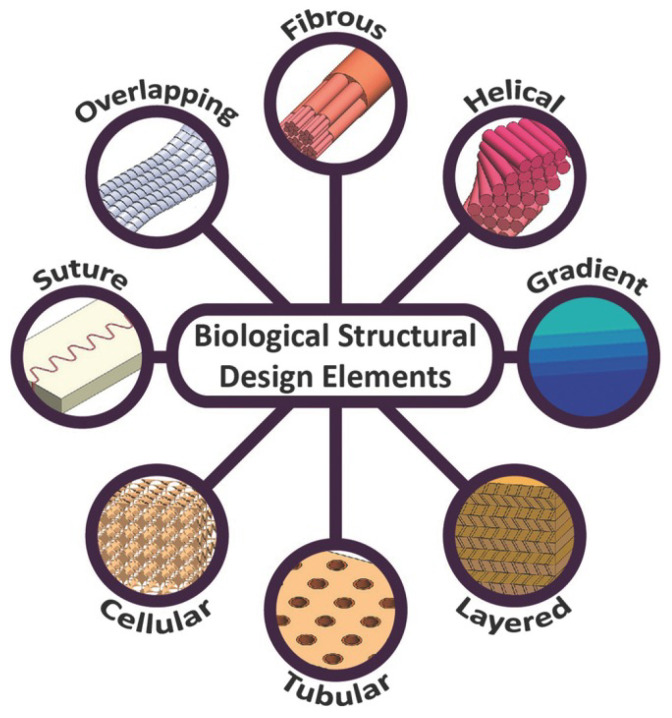
Diagram of the eight most common biological structural design elements. Adapted with permission. Ref. [48], 2015, John Wiley and Sons.

These composite materials furthermore display a hierarchical structural organization of their constituent materials across scales, from the nano-scale to the macro-scale [40,41,42,43,44,45,46,47]. In their article “Structural Design Elements in Biological Materials: Application to Bioinspiration”, Naleway et al. (2015) [48] outline the most common biological structural design elements found in nature’s hierarchical materials (Figure 2). Although different biological materials utilize various combinations of these design elements, along with varying ratios of hard to soft materials and organic to non-organic components, the common thread among them is that their hierarchical composite strategy enhances their strength, lightweight properties, toughness, and resistance to impact through encouraging localized rather than catastrophic material failure [40,41,49,50,51,52,53,54,55].

For instance, the intricate brick-and-mortar layered structure of nacre contributes to the exceptional robustness and fracture toughness of mollusk shells through toughening mechanisms such as crack deflection, fiber pull-out, organic matrix bridging, and molecular toughening [56,57,58,59,60,61,62,63,64].

Another example is the Bouligand structure, characterized by helicoidal arrangements of fibrous layers found in various organisms such as the mantis shrimp’s dactyl club, crab exoskeletons, and the scales of ancient fish like Coelacanth [65,66]. These structures exhibit outstanding toughness and impact resistance due to their ability to deflect cracks and dissipate energy. Computational simulations have further confirmed that the helicoidal layering, particularly in the double-twisted configurations found in certain fish scales, enhances inter-laminar strength and mitigates delamination [65]. This unique material architecture controls crack propagation through mechanisms such as crack twisting and, similar to nacre, limits the sensitivity of these biological structures to local failure [65,66].

Nature’s strategy of employing hierarchically structured composite materials to produce lightweight materials with remarkable mechanical properties and great functional variability, considering the weak constituents from which they are assembled [67,68,69,70,71], has provided valuable insights for engineering materials that mimic the structural and mechanical characteristics of their natural counterparts [69,71,72,73,74,75,76,77,78,79,80]. These naturally occurring hierarchical composites have served as archetypes for the development of man-made composites that exhibit superior toughness, low weight, and efficient energy absorption properties [38,39,54,78,79,81,82,83,84,85,86,87,88,89,90,91,92,93,94,95,96,97,98]. Potential applications for these bio-inspired engineered composites have been thoroughly discussed and have been successfully implemented in different fields [46,68,78,84,86,87,88,91,93,99,100,101].

**Figure 3:**
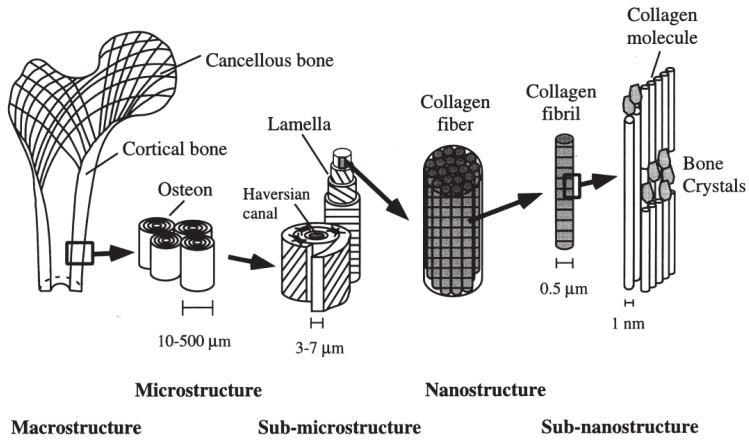
The hierarchical structure of bone. Adapted with permission. Ref. [113], 1998, Elsevier.

Hierarchical composite structures are also evident in human bone, spanning multiple scales of organization. This hierarchy encompasses the nano-scale arrangement of hydroxyapatite crystals intertwined with collagen fibrils, the staggered pattern of collagen fibrils themselves, evident at the micro-scale, and extends to the macroscopic level, where it is concluded in the composite composition of compact cortical and spongy trabecular bone [102,103] (Figure 3). The trabecular bone’s material properties, such as its viscoelasticity, allow it to absorb and dissipate energy. Moreover, the hierarchical arrangement of relatively weak materials at various scales further contributes to the exceptional toughness and fracture resistance of the final bone structure, all while retaining its lightweight characteristics [16,62,104,105]. The structure of bone has inspired the development of new synthetic materials that exhibit remarkable crack resistance, toughness, and lightweight properties [62,106,107,108,109,110,111,112].

Recently, Stagni et al. (2024) [112] were inspired by the micro-structure of bone, particularly the osteon-like structures, to develop a novel design for fiber-reinforced composites. By mimicking the concentric lamellae and Haversian canals found in bone, the researchers created multi-layered osteon-like structures embedded within a laminate structure. Their work demonstrated that these bio-inspired designs significantly improved the composites’ fracture toughness and damage tolerance. Specifically, they found that the osteon-like structures acted as effective crack deflectors, increasing the toughness of the material by up to 26% compared to traditional laminates, all while maintaining comparable stiffness and strength.

## 3. Cellular Structures

**Figure 4:**
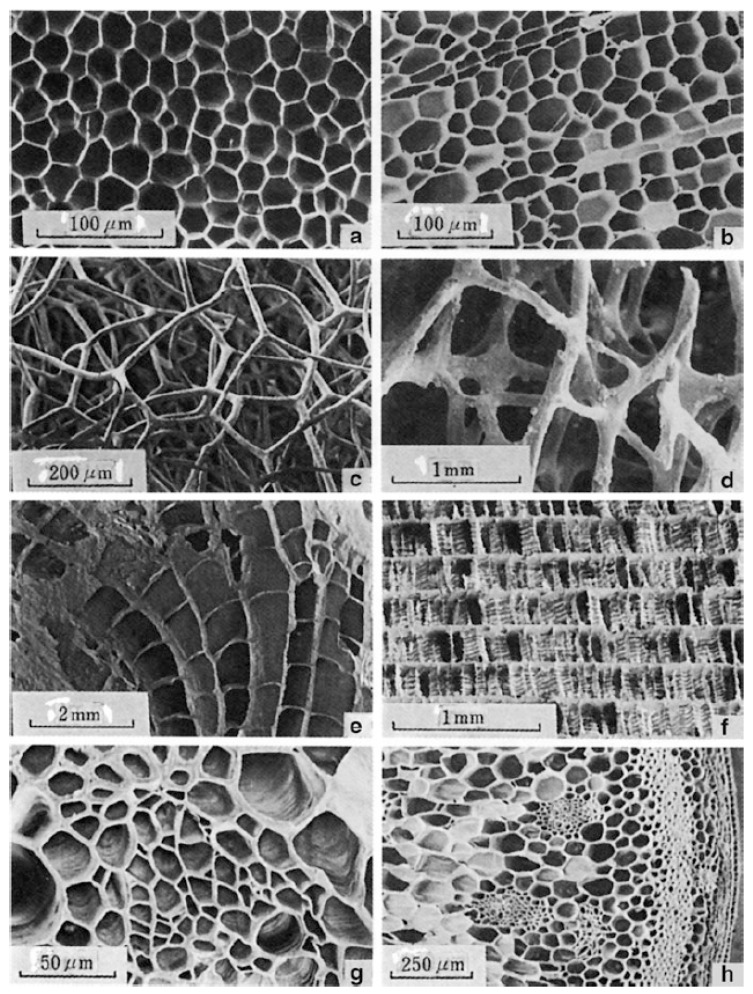
Natural cellular materials: (**a**) Cork, (**b**) balsa wood, (**c**) sponge, (**d**) cancellous bone, (**e**) coral, (**f**) cuttlefish bone, (**g**) iris leaf, and (**h**) stalk of a plant. Adapted with permission. Ref. [114], 1997, Cambridge University Press.

One strategy that has independently evolved in several instances in nature and is observed across multiple length scales is the use of cellular structures, which is evident in natural foams (Figure 4). Natural cellular solids take on various forms, ranging from honeycomb structures with prismatic cells in cork and wood to interconnected ligaments in sponges and cancellous bone, to the round cellular, and occasionally tubular, networks found in plant leaves and stems.

Natural foams represent nature’s strategy for creating less dense solids, effectively reducing material usage and overall weight while maintaining geometric integrity and adequate stiffness [115].

Typically, nature does not rely solely on cellular solids but combines hierarchical materials with cellular structures to achieve optimal mechanical properties. However, Yang et al. (2022) [116] found that certain organisms, such as the echinoderm *Heterocentrotus mamillatus*, rely heavily on the geometry of their cellular solids, composed primarily of magnesium-doped calcite, to achieve remarkable mechanical performance. The stereom’s bicontinuous cellular structure, featuring negative Gaussian curvatures and interconnected branches, minimizes stress concentrations and enhances both strength and damage tolerance. The study revealed that under mechanical stress, this structure facilitates microfracture and local densification, where fractured fragments jam into small openings, forming damage bands that absorb significant energy. This geometry-driven design allows the stereom to achieve a high level of resilience, illustrating how nature can effectively use geometric design to create robust structures, even with materials that are not inherently tough.

Researchers and engineers have successfully designed and produced bio-inspired foams and lattice structures demonstrating impressive specific strength and stiffness [117,118,119,120,121,122,123,124,125]. Engineered cellular solids have especially gained popularity in biomedical [126,127,128] and aerospace [129,130,131] applications. In the biomedical field, these structures help in adjusting the stiffness of medical implants to more closely match that of human bone. This adjustment reduces the risk of stress shielding while allowing fluid flow due to their inherent porosity [126,128,132,133,134]. In the aerospace industry, where reducing aircraft weight is crucial for minimizing fuel consumption and environmental impact, the high strength-to-weight ratio of lattice structures is particularly valuable [129,131,135,136].

Another distinct type of periodic structure found in nature is the biomineralized skeletal frameworks observed in certain sea sponges and marine microorganisms, such as Radiolaria. Unlike natural foams that occupy three-dimensional space within an organism, these lattice structures create an intricate outer shell. Additionally, periodic structures in nature can take the form of ribbing and venation patterns, found in plant leaves, insect wings, and the surfaces of some seashells, which typically combine branching with periodic patterns. Beyond functions like buoyancy, fluid permeability, and nutrient delivery, these cellular arrangements also serve structural and mechanical roles. For instance, periodic ribbing and venation reinforce surfaces that need to be large for functions such as maximizing sunlight exposure in leaves and enabling efficient flight in wings, without significantly increasing weight [137,138,139,140,141].

Skeletal frameworks in natural structures, along with ribbing and venation patterns observed in biological surfaces, have significantly inspired the development of analogous patterns to reinforce engineering surfaces [142,143,144,145,146,147]. Recently, Lin et al. (2024) [142] developed a design and optimization method for sheet reinforcement, directly inspired by the Voronoi patterns found in dragonfly wings. By applying the structural principles observed in these natural patterns, they successfully created lightweight reinforced sheets with improved bending resistance and vibration stability.

Beyond lightweight stiffness, in organisms and body parts vulnerable to impacts, the inherent flexibility of cellular solids, particularly those with low relative densities, provides a cushioning effect, thereby improving impact absorption through efficient energy dissipation. Cellular solids found in pomelo peels, cancellous bone, and plant stems have been mimicked to produce bio-inspired foams and lattice structures with remarkable impact absorption properties [148,149,150,151,152,153,154,155,156,157,158,159,160,161,162]. These cellular solids have been utilized in various collision-resistant structures and protective gear [163,164,165,166].

In a recent study, researchers developed a hierarchical lattice inspired by the skeletal system of glass sponges. By integrating bio-inspired features such as double diagonal reinforcement and hierarchical circular modifications, they designed a modified face-centered cubic lattice that demonstrated high strength, exceptional energy absorption, and enhanced damage tolerance. The design approach combined the benefits of both tensile-dominated and bending-dominated lattices, resulting in a material that outperformed traditional lattice structures in terms of specific strength and stability, while significantly reducing the risk of catastrophic failure [122].

Furthermore, in current-feeding organisms like corals and sponges, a cellular structure enables efficient expansion over a larger volume with less material. This increases the feeding area while maintaining structural integrity against underwater currents. Bio-inspired cellular solids with similar properties have been used in the biomedical field to produce bone implants with a large surface area to facilitate cell attachment and growth [126,132,133,134], in catalytic converters and reactors to increase the surface area available for catalytic reactions [167,168,169,170], and in heat exchangers to increase the surface area for heat transfer [171,172,173,174].

Additionally, a common characteristic of nearly all natural periodic structures, such as those depicted in Figure 4, is the presence of geometric irregularities. Efforts to quantify this structural disorder in nature have been made [175,176,177], and analyses suggest that introducing such irregularities enhances stiffness, fracture toughness, and vibration properties in analogous engineered periodic structures [175,177,178,179]. Finally, some studies have shown that components composed of cellular structures are less sensitive to flaws. Compared to their solid counterparts, these structures retain a higher level of stiffness when damaged, partly by ensuring that alternate load paths remain available even after damage occurs [116,180,181,182].

Apart from alternate load paths, another key strategy in nature that enhances structural robustness is compartmentalization. This approach involves creating physical or functional discontinuities within a structure to prevent damage from spreading beyond the initial failure zone. For example, some plants, like the lilium auratum, use segmented seed pods to limit damage to specific compartments, ensuring the survival of the rest of the structure [183]. This bio-inspired strategy has been particularly valuable in civil engineering and the design of large structures, where it enhances robustness by isolating failures and minimizing their impact on the overall system.

Biagi (2015) [184] explains how structural complexity, defined as the degree of interaction between load paths, is enhanced by compartmentalization. While alternate load paths provide redundancy, compartmentalization isolates failures to reduce their impact on the overall structure, significantly increasing robustness, especially under extreme conditions.

Kiakojouri et al. (2023) [183] highlight how bio-inspired compartmentalization strategies have already been successfully applied in various engineering contexts. For instance, construction joints are used in buildings to create intentional discontinuities that help contain damage. Similarly, in large bridges, expansion joints allow different segments to move independently, preventing the spread of damage due to thermal expansion or seismic activity. Huber et al. (2019) [185] also discuss how compartmentalization techniques, like segmenting large structural components, have been used to ensure the safety and longevity of timber buildings.

Alternate load paths and compartmentalization underscore a fundamental principle of natural structures: prioritizing resilience and adaptability over singular optimal performance, thereby ensuring reliable functionality across a range of unpredictable conditions.

## 4. Functional Gradients

**Figure 5:**
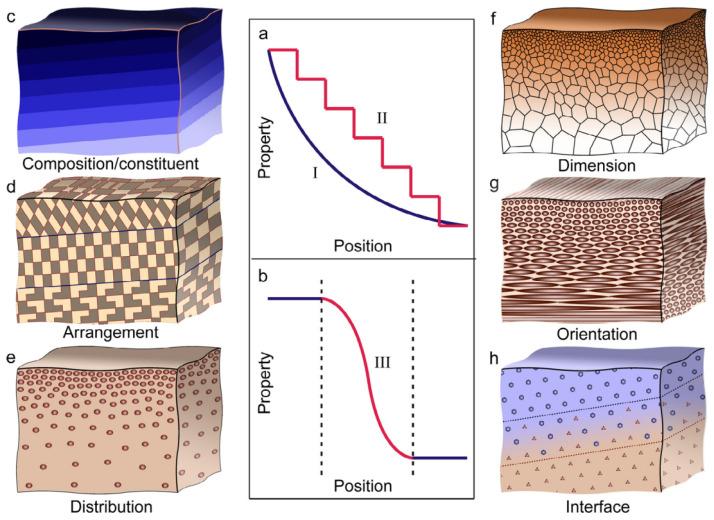
Local property profiles and basic forms of gradients in biological materials: (**a**) Local properties change either gradually (I) or in a step-wise manner (II) through the entire material volume. (**b**) Local properties vary continuously (III) across the interface between dissimilar components. (**c**–**g**) The gradients in biological materials are fundamentally associated with changes in chemical composition/constituents (**c**) and structural characteristics, including the arrangement (**d**), distribution (**e**), dimensions (**f**), and orientations (**g**) of building units. (**h**) Gradient interface in biological materials. Adapted with permission. Ref. [186], 2017, Elsevier.

Another structural strategy observed on multiple scales in nature is the use of functional gradients. In this context, the word ‘gradient’ refers to the gradual, spatial transitions observed in natural materials and structures. These transitions appear in various forms, and different instances may involve changes in chemical compositions, micro-structures, geometries, or any combination of these. Furthermore, these gradients are evident within regions of a single material as well as at the interfaces between distinct materials or structural elements. Liu et al. [186] schematize the basic forms of gradients in biological materials and structures in Figure 5.

Functionally, nature utilizes heterogeneity in composition and structure within its components to minimize material use, tailor local properties, and often achieve multi-functionality. The gradual and seamless nature of this variability, as opposed to being abrupt, allows for a more efficient attainment of these goals.

In their natural environments, biological components are exposed to diverse structural loads. These loads would typically result in non-uniform stress distributions if the components were made of homogeneous material. Nature addresses this by allocating more material to heavily loaded regions and less to lightly loaded areas. However, abrupt changes in a material’s mechanical properties, such as stiffness, hinder the smooth transfer of stress, leading to stress accumulation at boundaries between regions with differing material properties. High-stress points can negatively affect a component’s static loading performance and create conditions favorable for crack initiation and fatigue failure, undermining the advantages of nature’s material and structural heterogeneity. To counter this, nature employs smooth spatial gradients to transition from stiffer to softer materials, reducing the risk of material failure [88,187,188]. A recent study investigated the effect of gradient design on mechanical performance using porous 3D-printed models with bio-inspired continuous gradients [189]. The results demonstrated that both the magnitude of the gradient and its continuity significantly influence performance. By introducing a continuous and large gradient, the maximum flexural load and energy absorption capability increased substantially compared to models with sharp gradients or no gradient.

Human bone is an excellent illustration of natural gradients, in various forms and across multiple scales, serving efficient load distribution. In trabecular bone, the composition and micro-structural arrangement of the constituent material, and consequently, its density and stiffness, change gradually across different areas [190]. On a larger scale, within areas consisting solely of trabecular bone, the volume fraction of this natural cellular structure varies continuously from 0.05 in more porous regions to 0.6 in denser regions [190]. This variation is achieved through changes in the distribution, dimension, and orientation of the cells that make up the cellular solid. Furthermore, the transition from the very porous cancellous bone to the much denser cortical bone, each with distinct micro-structural compositions, is quite gradual [191], exemplifying nature’s strategy of utilizing gradients at interfaces of dissimilar materials.

**Figure 6:**
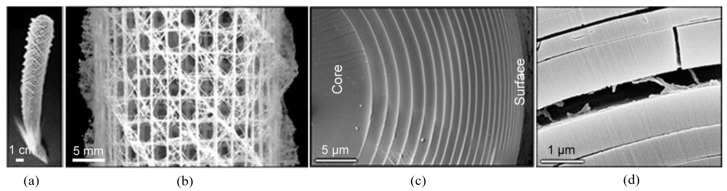
The skeletal structure of *Euplectella* sp. (**a**) An image of the complete skeleton; (**b**) a piece of the skeleton with helical reinforcing ridges; (**c**) an SEM image of a cross-section through a standard spicule within a strut, displaying its graduated laminated design; and (**d**) an SEM image of a broken spicule, exposing an organic layer in between. Adapted with permission. Ref. [192], 2005, The American Association for the Advancement of Science.

Apart from facilitating efficient load distribution, some natural structures incorporate dimensional gradients to enhance toughness. Marine sponges (Figure 6a) are a prime example. The sponge’s porous skeleton (Figure 6b) includes spicules for structural reinforcement. These spicules consist of a core of hydrated silica surrounded by successive layers of silica and protein-based materials. The thickness of these silica layers decreases from the inner core to the outer surface, as illustrated in Figure 6c. This gradation in layer thickness is crucial in controlling crack propagation since cracks predominantly follow the organic layers (Figure 6d). The outer, thinner layers restrict the depth of crack penetration, while the inner, thicker layers contribute to overall stiffness [192,193]. The gradual, layered arrangement of silica rings within the sponge’s spicules is a prime example of how nature combines multiple strategies—specifically compartmentalization and functional gradients in this case—to efficiently achieve enhanced, more complex mechanical performance.

Furthermore, by tailoring local properties with spatial gradients, nature crafts multi-functional components using a limited number of compositional building blocks. For instance, a gradual transition from a soft interior to a stiff and hard exterior maintains the overall stiffness and wear resistance of the component, while also facilitating additional functions, such as energy absorption. This is particularly advantageous in body parts subject to contact impact forces, such as tooth enamel, sheep horns, and horse hooves [38,48]. It has long been recognized that incorporating bio-inspired spatial gradients can greatly enhance the mechanical performance of man-made parts and materials. Consequently, engineered Functionally Graded Materials (FGMs) have been developed and are now used for various engineering purposes. These include resisting contact deformation and damage in engineering components [189,194,195,196], developing tough, wear-resistant, and energy-absorbent biomedical implants [132,133,134,197,198,199], and improving interfacial bonding between components made of dissimilar materials [163,200,201,202,203,204].

In a recent study, researchers combined the advantages of cellular structures and functional gradients to develop optimized lattice structures for orthopedic implants [132]. Drawing inspiration from the graded architecture found in natural bone and the performance benefits of cellular configurations, they designed a lattice with a gradient specifically tailored to enhance both mechanical and biomedical properties. By integrating a graded lattice unit cell with features such as high surface curvature and varying porosity, the design achieved superior bio-compatibility while minimizing stress shielding—a common issue in traditional implants. This approach highlights the value of integrating multiple bio-inspired strategies to fully harness the benefits of biomimetic design.

## 5. Hard Shell–Soft Core Architecture

As a matter of fact, a hard outer shell paired with an elastic core is a common feature in nature, serving mechanical functions beyond contact damage resistance and energy absorption. This arrangement is typically found in structures that need to withstand high loads from axial compression, bending, and torsion. In structural engineering, striking a balance between global deformations, such as bending, torsion, and buckling, and local deformations like local buckling or crimping, is essential. For instance, in a hollow cylindrical tube, optimizing stiffness against bending and global buckling while maintaining constant mass involves maximizing the moment of inertia by increasing the radius and minimizing the wall thickness. However, a thinner wall increases the tube’s vulnerability to local buckling [205].

**Figure 7:**
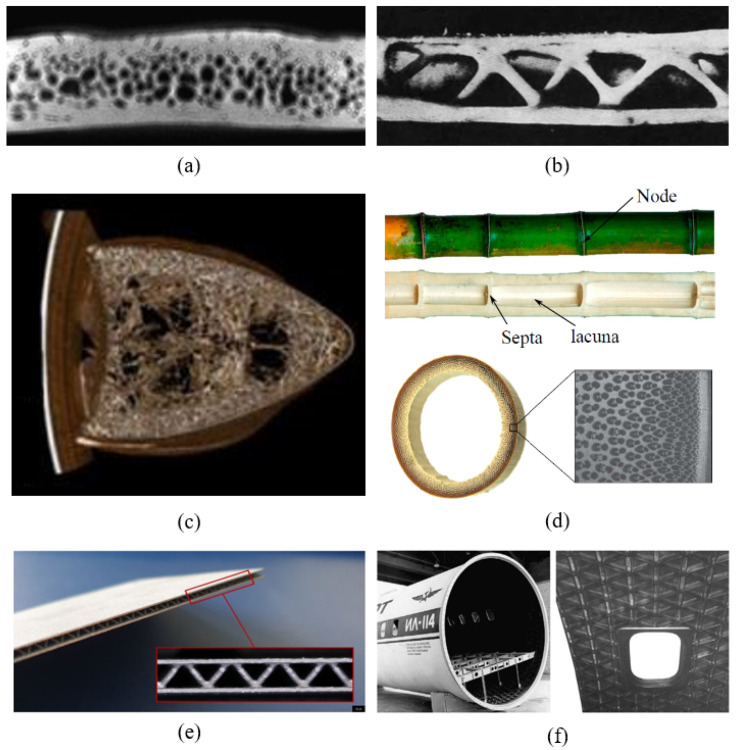
(**a**) A top-sectional view of a turtle shell carapace coupon obtained from a computed tomography (CT) single-slice scan showing randomly distributed closed cell pores within the foam-like interior layer. (**b**) Section of bone structure from a vulture wing [226]. (**c**) Toucan beak showing the porous interior (bone) with a central void region. (**d**) Cross-section of bamboo stalk showing periodic internal discs and radial distribution of fibers through the wall thickness. (**e**) Additively manufactured sandwich panel with pyramidal core [218]. (**f**) Carbon–epoxy lattice fuselage section and the window frame. (**a**) Adapted with permission. Ref. [227], 2009, Elsevier. (**c**) Adapted with permission. Ref. [209], 2013, The American Association for the Advancement of Science. (**d**) Adapted with permission. Ref. [228], 2019, Elsevier. (**f**) Adapted with permission. Ref. [213], 2006, Elsevier.

Nature addresses this by filling the core with natural foam, a feature observed in various structures: bone (Figure 3), plant stalks (Figure 4h), porcupine quills [206], turtle shells (Figure 7a), bird beaks (Figure 7c), and feathers [207]. Additionally, more hollow, truss-like cores are seen in bird bone structures such as vulture wing bones (Figure 7b). This strategy, in its various forms, significantly enhances natural structures’ resistance to local buckling while minimally increasing their weight [208,209].

In bamboo stalks, the core is segmented by disc-like reinforcements, which are orthogonal to their functionally graded cylindrical walls (Figure 7d). This combination of shell and cellular architecture, along with hierarchically arranged composites and multi-scale functional gradients, enhances load-bearing efficiency. Numerous studies have demonstrated that the structure of bamboo stalks increases their resistance to compression and bending failure, as well as to both global and local buckling [210,211,212].

The combination of shell and cellular solid composition has been utilized to develop lightweight solid–lattice hybrid structures used in various aerospace components [213,214,215], such as aircraft fuselages (Figure 7f), as well as bending-resistant sandwich panels with cellular cores (Figure 7e) [216,217,218].

Furthermore, sandwich panels, particularly those with bio-inspired core designs, have proven to be highly effective in enhancing structural performance under extreme loading conditions, such as impacts and blasts. Nature-inspired core architectures, like those mimicking honeycombs, bamboo, and beetle forewings, exhibit exceptional energy absorption due to their hierarchical designs. In their review paper, Ha and Lu (2019) [154] emphasize how bio-inspired sandwich panel cores leverage multiple natural design principles such as hierarchical structuring, functional gradients, and multi-cellular configurations to optimize energy absorption while maintaining lightweight properties. These hybrid designs outperform conventional configurations in blast mitigation by more efficiently distributing and dissipating energy [154,219].

Similarly, Guo et al. (2024) [220] and Birman and Kardomateas (2018) [221] highlight how hierarchical honeycomb cores and bamboo-like multi-tubular structures offer enhanced buckling resistance and energy dissipation by imitating natural gradients and composite layouts. The integration of features like multi-scale cellular cores, inspired by plant stalks or beetle forewings, enables these panels to manage both local and global deformations, ensuring overall structural integrity under both static and dynamic loading scenarios, including impact and blast conditions [222,223].

Bio-inspired sandwich panels are increasingly adopted in aerospace and transportation, where maintaining strength and impact resistance while minimizing weight is critical. These structures are employed in aircraft fuselages, automotive crash absorbers, and protective armor, benefiting from efficient load distribution and energy absorption [224,225].

## 6. Form Follows Function

Cellular structures, functional gradients, and architectures with hard shells and elastic cores all illustrate how materials and geometry in nature are influenced by the intended mechanical roles. The following natural strategies especially highlight how mechanical function shapes natural structures, demonstrating load-bearing principles that align with the phrase ‘form follows function’, first coined by architect Louis Sullivan in the early 20th century.

Nature aligns materials within its load-bearing structures to correspond with the direction of stress and force. An example of this is observed in plant cell walls. In these walls, stiff, semi-crystalline cellulose fibrils serve as the primary load-bearing elements [229]. These fibrils are interconnected by hemicellulose polymers and are embedded within a gel-like matrix of pectins, leading to the characterization of the cell walls as composite materials [230]. Research has demonstrated that the average orientation of the cellulose fibrils within the composite wall influences the wall’s growth anisotropy, and consequently, its mechanical properties [231,232]. Furthermore, it has been demonstrated that the mechanical strength of stems is largely attributable to the alignment of cellulose fibrils with the direction of stress. This stress primarily arises from the plant’s weight against gravity and bending due to wind forces [233,234]. Nature’s strategy of aligning stiff fibers within a compliant matrix in the direction of force to create lightweight structures with high load-bearing performance has repeatedly inspired the development of lightweight, man-made reinforced composites featuring fibers with optimized orientation, along with innovative methods for their efficient production [235,236,237,238,239,240,241,242,243].

For example, Heitkamp et al. (2023) [238] developed and investigated a method for embedding continuous fibers in 3D-printed parts by aligning them along principal stress directions determined through finite element analysis. The researchers demonstrated that generating fiber paths based on stress trajectories led to significant improvements in tensile and flexural strength. Specifically, specimens with optimized fiber paths exhibited a 3-fold increase in peak load during tensile tests and a 1.9-fold increase during flexural tests compared to those with traditional unidirectional fiber alignment.

**Figure 8:**
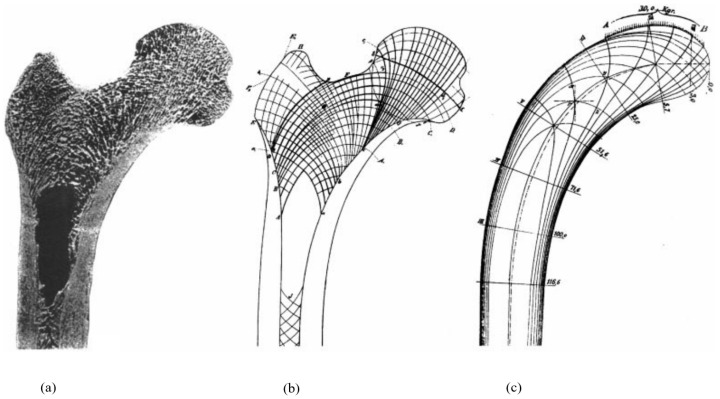
(**a**) Mid-frontal section of the proximal femur, showing trabecular architecture [254]. (**b**) Schematic representation of trabecular patterns [255]. (**c**) Principal stress trajectories in a comparable model subjected to a downward distributed load acting on a section of the top surface [256].

Trabecular bone offers another example of how nature aligns its materials with stress directions, resulting in components that are mechanically optimized at multiple length scales. Within the femur, trabeculae create a complex network of curving lines that stretch from the bone’s head to its shaft. These lines intersect regularly, often at right angles, forming an interlaced structure (Figure 8a,b). This pattern has been recognized as a direct representation of the principal stress lines (PSLs) arising from the load cases the femur typically encounters. Figure 8c shows the PSL diagram in a simplified geometry, analogous to the femur’s general shape and subjected to a similar load case, demonstrating a good match with the actual trabecular patterns. These lines represent the paths within a mechanically loaded material where the dominant stresses are purely axial—either tensile or compressive—and are devoid of shear stresses. The distribution of material along principal stress directions is widely acknowledged as an effective method for creating lightweight and load-bearing structures [244]. The structure of trabecular networks and the natural strategy of aligning material along principal stress lines have inspired the development of methods to produce optimized load-bearing truss structures following the same principles, along with various methods for their production [245,246,247,248,249,250,251,252,253].

A recent study [253] introduced a stress-driven infill mapping technique for 3D-printed continuous fiber composites, combining optimized truss-like infill with stress-aligned continuous fiber paths. By mapping fiber trajectories to principal stress directions and simultaneously tuning infill density, the approach enhances load-bearing efficiency and mechanical performance. The integration of both strategies resulted in structures with significantly improved stiffness and strength compared to conventional patterns, demonstrating the effectiveness of aligning both fiber and infill along stress paths.

In fact, the trabecular patterns within bones are dynamic, adapting to the loads experienced during skeletal activity [226]. This plasticity in bone structure is driven by mechanotransduction, a process governed by osteocytes, involving the remodeling of bone [257]. This not only entails the realignment of trabecular material along principal stress directions but also includes the deposition of new bone in response to increased stress in some regions and the resorption of bone under reduced stresses in others. Consequently, this leads to regions of dense trabecular bone, areas with more porous bone, and even void regions where stress is minimal or non-existent. This phenomenon is evident in the femur, as illustrated in Figure 8a, and in the core of the toucan’s beak, shown in Figure 7c. The hollow center of the toucan’s beak, for instance, indicates exposure to bending forces during activities such as feeding and preening, which would produce little stress near the center [209].

Similarly, plant and tree morphogenesis is heavily influenced by external physical forces. Studies have shown that these forces lead to the remodeling of plant tissue [258], which is reflected in changes in mechanical properties in response to the load [259]. In some cases, this is evident in alterations of leaf venation patterns and thickness [260].

**Figure 9:**
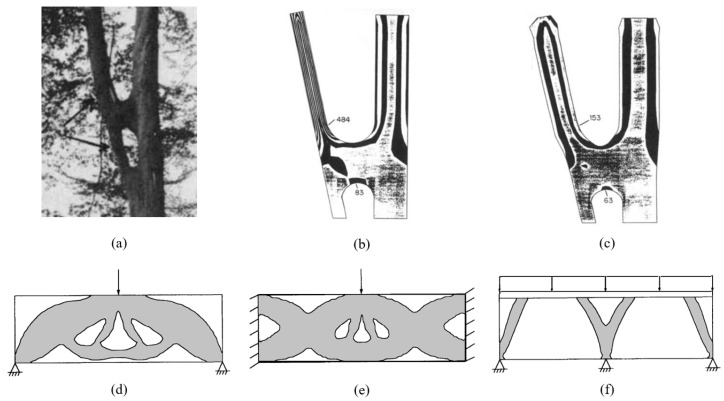
(**a**–**c**) Mattheck’s shape optimization based on biological growth. (**a**) Framework tree showing further increase in diameter only above the lateral bridge. (**b**) Isolines of the von Mises stresses before computer-simulated growth. (**c**) Same as (**b**) but after growth. (**d**–**f**) Application of SKO to problems with different loading conditions: (**d**) Simply supported with single central force. (**e**) Both ends clamped with single central force. (**f**) Three points supported and predefined top plate under constant pressure. (**a**–**c**) Adapted with permission. Ref. [264], 1990, Elsevier. (**d**–**f**) Adapted with permission. Ref. [266], 1992, Elsevier.

Trees, on the other hand, develop reaction wood in response to mechanical stresses resulting from natural forces such as wind and gravity, adding material to reinforce branches [261]. Furthermore, this addition of material is strategically executed to maintain geometric continuity in the overall structure, thereby avoiding sharp edges. This principle is exemplified by the arched geometry that seamlessly connects tree branches to each other and to the trunk (Figure 9a), as well as the root flare—the filleted region that smoothly transitions the trunk to the ground and tree roots. Such smooth geometric transitions between different structural elements are crucial for efficient stress transfer. They prevent stress hotspots that can arise from tangential discontinuities and sharp edges, known as notch stresses [262]. This is similar to how stiffness discontinuities create stress concentrations in materials, as previously discussed.

The concept of ‘organic’ shapes in structural design, characterized by smooth contours and junctions aimed at enhancing structural load-bearing efficiency, is influenced by nature’s strategic use of such forms in its various load-bearing structures. Furthermore, nature’s optimization of material distribution to create load-efficient structures with minimal material has inspired the development of similar structural optimization methods in engineering. The German scientist Claus Mattheck pioneered the study of geometric design and growth ‘rules’ in biological structures, particularly trees. He applied his findings to develop design principles and shape optimization methods in engineering [263,264,265]. For instance, in his article “A new method of structural shape optimization based on biological growth” [264], Mattheck introduces a geometric method for reducing notch stresses, inspired by growth patterns of tree wood (Figure 9b,c).

Another early method developed in this field of structural optimization was the soft kill option (SKO), a technique closely influenced by mechanotransduction in bone. The SKO involves iteratively removing material from low-stress areas in a loaded structure, thus producing designs that focus material usage where it is most needed for load-bearing purposes [266]. Figure 9d–f present structures derived from applying SKO to bodies under various example loading conditions. Since its inception, the field of structural optimization has evolved significantly, with the development of new, highly efficient methods in recent decades [267,268,269].

Finally, nature excels at simultaneously optimizing material and geometry across multiple scales. Diatoms, a class of aquatic autotrophic microorganisms characterized by silicified exoskeletons with highly complex architectures, are an exemplary case of how material properties and multi-scale structural geometry in nature work together to influence mechanical behavior [270]. The frustules of diatoms are primarily composed of amorphous silica, intricately combined with organic molecules to form a composite material. This composite structure enhances the toughness and flexibility of the frustules, as the organic components help arrest cracks and prevent catastrophic failure [271,272]. The synergy between these organic and inorganic components allows diatom frustules to maintain structural integrity under various mechanical stresses. Beyond their material composition, diatom frustules exhibit an intricate hierarchical geometry that spans multiple scales. At the nano-scale, organized pores reduce the weight of the frustule while preserving its strength by effectively distributing mechanical stresses [270,272]. Meso-scale features, such as ribs and other structural elements, provide additional support and contribute to the overall stiffness of the frustule [270,271,273]. On a macroscopic level, the frustules’ overall shape, including features like the raphe, optimizes lightweight strength and stiffness [273].

Features inspired by diatom shells have significantly enhanced the performance of engineering components when applied, and have also driven the development of structural optimization methods for various mechanical objectives [124,146,147,274,275,276,277].

For example, Linnemann et al. (2024) [146] were inspired by the frustule structures of diatoms to develop stress-adaptive stiffening structures for lightweight surfaces. By abstracting the morphological features of diatom frustules, they designed parametric models that optimized bending stiffness in engineering surface components. The study revealed that these diatom-inspired structures achieved a significant decrease in displacement compared to a conventional reference model with equivalent mass.

Moreover, recent efforts have focused on developing methods to optimize engineering composite material parts across multiple length scales concurrently [240,278,279,280,281,282,283].

For example, Ren et al. (2024) [240] introduced an approach to simultaneously optimize structural topology and continuous fiber tool paths in additive manufacturing. Unlike conventional methods that treat topology optimization and fiber tool path design as separate steps, this integrated framework considers both processes concurrently. In this way, the fiber paths are precisely aligned with the principal stress directions while optimizing the material layout within the structure. This concurrent optimization significantly improves load distribution and overall stiffness, leading to better material utilization and more efficient fiber paths compared to traditional sequential optimization methods.

Similarly, Ichihara and Ueda (2023) [278] introduced a hybrid optimization strategy that combines topology optimization with locally latticed truss structures to enhance toughness in 3D-printed short-fiber-reinforced composites. The study employed anisotropic topology optimization, utilizing intermediate material fractions to strategically introduce lattice regions. These lattices were optimized for both material distribution and toughness by promoting local buckling mechanisms under load. The design effectively combines high stiffness with enhanced toughness, as demonstrated through three-point bending tests that showed significant improvements in residual toughness compared to conventional designs. This approach further underscores how integrating multiple nature-inspired structural strategies—at multiple length scales—can result in the most effective and robust engineering solutions.

## 7. Robust Geometric Shapes

The distinct trabecular patterns in the femur head and the shapes of tree wood are prime examples of natural geometries tailored to specific, rather expected mechanical loads. However, when confronted with less predictable load cases, nature often adopts more universally robust geometries. This shift in strategy is exemplified by the design of bamboo stalks, which utilize a hollow tube structure. This configuration offers resilience against bending forces from various directions [226].

In extreme scenarios, where the nature and direction of the load are highly unpredictable, such as impacts to the skull or eggshell, nature favors shapes like ellipsoids. The ovoid geometry of eggshells and the rounded, dome-like structure of the human skull are ‘designed’ to offer protection against impacts from multiple, unforeseen directions by evenly distributing stress across their surfaces. While robustness remains the central theme, in situations where even local failure could compromise the function of the biological component, nature employs a strategy that contrasts with hierarchical material architectures or compartmentalization, which localize failure. Instead, in these cases, nature’s approach is to spread the load across the entire structure, thereby decreasing the likelihood of even local failure. Ellipsoidal geometries, like those observed in the human skull, have been utilized in the design of protective gear and various pressure vessels.

This principle of employing regular, symmetric shapes for protection and strength under uncertain load conditions is also evident in other natural forms. For example, some seed coats exhibit a nearly perfectly spherical shape, providing strength and resistance against pressures from various angles, turtle shells utilize a dome shape, while many marine animals, such as snails, have adopted conical shells to achieve similar protective benefits. Other regular shapes in nature that exhibit intrinsic strength and robustness include the hexagonal honeycombs found in bee hives, and minimal surfaces like gyroids, observed in butterfly wings and underwater current-feeding animals such as bryozoa.

Furthermore, like all natural strategies, these geometries do not appear in isolation; instead, multiple features at varying scales work in unison to achieve the intended function most efficiently. For example, in the human skull, it is not just the general geometry but also the spatially variable thickness and material properties of the cranial bone that together provide the skull with its impact protection properties [284,285].

## 8. Challenges and Future Needs

Translating natural structures into functional engineering components generally involves several foundational steps, including characterization, modeling, optimization, and manufacturing. Characterization is typically the initial critical step, where the intricate features and properties of natural materials and structures are thoroughly analyzed. Due to the inherent complexity of biological structures, direct one-to-one modeling is often infeasible, necessitating abstraction. This process distills the natural system to focus on the aspects most relevant to engineering objectives and produces simplified models that aim to accurately reflect the properties of the biological archetype. Moreover, since different engineering components must perform specific functions and withstand varying load cases, it is often necessary to manipulate and optimize the computational model. For example, a parametric definition of the bio-inspired model can be established, allowing for the optimization of parameters such as cellular solid density to achieve lightweight strength under specific load conditions. Finally, the designs generated from the previous steps must be manufacturable. Each of these steps presents its own set of challenges.

Characterizing natural materials and structures, especially at smaller scales, is particularly challenging due to their inherently complex hierarchical organization and multi-scale interactions. Traditional characterization techniques often fall short in capturing the full scope of these intricacies.

However, recent advancements in characterization approaches and technologies have begun to address this challenge. Micheletti et al. [286] highlight the potential of a correlative approach that combines X-ray tomography, X-ray scattering, vibrational spectroscopy, and atom probe tomography with electron microscopy. This strategy would offer valuable insights into the diverse levels of organization within nature’s hierarchical materials, such as bone. Additionally, synchrotron-based techniques have recently provided unprecedented insights into the hierarchical organization of natural materials [287]. These multi-scale structural characterization methods have enabled the visualization and analysis of complex interactions across different scales, from the nanometer to the millimeter range, which is essential for understanding the structure–function relationships in hierarchical materials. Furthermore, Holm et al. (2020) [288] discuss how machine learning and computer vision techniques are revolutionizing micro-structural characterization. Their overview highlights how convolutional neural networks and other machine learning algorithms can automate the analysis of micro-structural images, extracting high-dimensional data and enabling the discovery of new metrics and trends that were previously inaccessible with traditional methods.

Another significant challenge in replicating natural structures in engineering designs is the difficulty of accurately and efficiently modeling these complex systems. Even with advanced characterization, the intricate and multi-scale nature of natural systems often necessitates simplifications and abstractions. However, if not carefully managed, these abstractions can result in the loss of critical functional details, making it challenging to fully replicate the mechanical properties and responses of natural structures in engineered designs.

Despite these challenges, advancements in computational methods, particularly those involving generative design and artificial intelligence, are beginning to overcome these obstacles [283,289,290,291,292,293,294,295,296,297]. For example, Buehler (2023) [293] introduced a computational approach leveraging a deep neural network to efficiently model complex hierarchical micro-structures. Similarly, the development of representative volume elements for nacre by Leuther et al. (2023) [291] has enabled the detailed simulation of its micro-structure. Lu et al. (2023) [289] utilized generative design coupled with deep learning techniques to model and design bio-inspired heterogeneous hierarchical spider web structures, successfully synthesizing complex 3D webs with diverse mechanical properties. In another recent study, Park et al. (2023) [298] employed a deep learning approach to accelerate the design of composite materials and uncover previously unexplored composite configurations [298].

Furthermore, to achieve its remarkable multi-functionality and efficiency, nature consolidates multiple functions within a single structure, often utilizing material combinations across multiple scales concurrently. In contrast, state-of-the-art engineering design and optimization tools face significant challenges when attempting to optimize for multiple criteria across different scales and materials simultaneously. These processes are computationally intensive and often infeasible, particularly because certain simulations—such as those evaluating dynamic behaviors like energy absorption, vibration analysis, or thermal–fluid interactions, as well as complex phenomena like crack propagation—are themselves extremely resource-demanding. As a result, the most widely applied structural optimization methods for load-bearing components predominantly yield designs with uniform material properties, features confined to a limited range of length scales, and optimal performance under only a very specific load case. This approach contrasts sharply with nature’s integrated and multi-functional strategies.

The limitations of current engineering design technologies are well known, and researchers are actively working to enhance their efficiency. For example, the development of simplified models that still accurately capture the mechanical responses of biological structures, as discussed in the previous paragraph and described by Buehler [293] as ‘computational building blocks’, is essential for reducing the computational effort required to integrate complex materials and structures into engineering design workflows. For instance, Feng et al. (2023) [294] developed a computational approach that generates simplified models of cellular structures, which can then be integrated into engineering design workflows. Numerical homogenization methods have also been extensively developed to facilitate the integration of heterogeneous media, such as composite and cellular structures, in engineering design [299,300,301,302,303,304].

Moreover, concurrent optimization across multiple scales has been a rapidly advancing topic in recent years, producing remarkable results, as highlighted in studies mentioned earlier [240,278]. Lee et al. (2024) [280] recently provided an extensive review of data-driven multi-scale design and optimization methodologies, emphasizing the potential of machine learning in multi-scale component design due to its superior ability to uncover the complex relationships between properties and geometries. Multi-material optimization is also an area of ongoing development, with continuous advancements being made [305,306,307].

Another significant recent development making computationally intensive engineering design simulations and optimization workflows more feasible is the advent of AI-assisted methods, which have become increasingly effective for accurately predicting complex physical phenomena [280,308,309,310,311,312,313,314,315,316,317,318]. These methods often employ mathematical approximations that function as surrogate models, replacing complex explicit mathematical equations to significantly reduce computational demands. One particularly impactful advantage is their ability to make multi-objective optimization problems—especially those requiring traditionally computationally intensive simulations—more feasible. For example, Zhang et al. (2023) [308] applied a machine learning approach that combined artificial neural networks with a genetic algorithm for the multi-objective optimization of heat exchangers. By using CFD simulation data to train the neural networks, they were able to predict the heat transfer coefficient and pressure drop with high accuracy in significantly less time, which enabled the feasible optimization of key heat exchanger design variables such as inlet air velocity and tube ellipticity. Similarly, Faraz et al. (2023) [318] utilized artificial neural networks for the multi-objective optimization of horsetail-inspired sandwich tubes to enhance crashworthiness. Their approach accurately predicted critical parameters such as peak crushing force and specific energy absorption, facilitating the optimization of geometrical features like tube thickness and core number.

Finally, efficiently designing multi-scale, multi-material components in digital environments is only one part of the challenge. The ultimate goal is to translate these designs into physical reality, yet manufacturing remains a significant bottleneck. In nature, the formation of structures involves bottom-up self-assembly processes that span from the nano-scale to the macro-scale. To fully unlock the potential of biomimetics in load-bearing engineering components, manufacturing techniques capable of reproducing such multi-scale configurations are crucial. A significant challenge in manufacturing bio-inspired structures lies in the difficulty of achieving precise control over composition, gradients, interfaces, micro-structures, and morphology [319]. Furthermore, despite the higher energy costs associated with producing synthetic composites, their combination of stiffness and toughness still often falls short compared to natural materials with similar compositions [78]. This shortfall can largely be attributed to the lack of advanced multi-scale manufacturing methods and the limited control over micro-structure and local composition in synthetic materials [320]. However, researchers are increasingly addressing these limitations through innovative manufacturing techniques that more closely replicate nature’s processes.

Additive manufacturing processes have seen remarkable advancements in recent decades, with some achieving the production of complex multi-material parts. For example, Ahn et al. (2024) [321] introduced a novel filament-based additive manufacturing method that allows for precise control over material composition and distribution, enabling the creation of complex multi-scale structures. By blending different materials within a single filament, they were able to fabricate objects with highly tunable properties, such as mechanical strength and electrical conductivity, all within a single printing process

Manufacturing techniques that harness self-assembly principles have also been gaining traction [322,323,324,325,326]. For example, Zhao et al. (2022) [326], inspired by the bio-mineralization process of enamel, developed an artificial enamel with a hierarchical structure across multiple length scales using a self-assembly pathway. Their method successfully replicates the atomic, nano-, and micro-scale structures found in natural biomineralized materials. Nano-indentation tests revealed that the Young’s modulus and hardness of the artificial enamel even surpassed those of natural tooth enamel.

Moreover, manufacturing methods that incorporate living cells have also seen significant development [320,327]. For instance, Xin et al. (2021) [327] utilized living cells to grow materials with predefined micro-structures. They combined bacteria with 3D-printed frameworks to create bio-inspired mineralized composites with ordered micro-structures. These composites demonstrated remarkable specific strength and fracture toughness, comparable to those of natural materials, along with superior energy absorption capabilities that exceed both natural and synthetic counterparts.

As demonstrated, significant advancements have been made in multi-scale material characterization methods, in the efficient modeling of complex structures, and in the development of feasible multi-scale and multi-material design and optimization techniques. Progress has also been achieved in the efficient production of these complex structures. However, the full potential of bio-inspired load-bearing strategies in engineering design is far from being fully realized. Continued efforts in line with the studies discussed in this section are essential to ultimately enable the creation of components that replicate the complexity and functionality of biological structures. Furthermore, accelerating this progress will require increasingly leveraging advancements in computing, particularly in AI.

From a wider lens, greater interdisciplinary collaboration is needed, combining insights from biology, materials science, structural engineering, and advanced computing to enhance bio-inspired engineering methods. It is also essential to adopt a system-level perspective in engineering design that reflects the integrative approach found in nature. A nature-inspired strategy promotes a holistic, bottom-up approach, building complexity from the ground up. This ensures that each component not only fulfills a specific function but also significantly enhances the overall cohesion and effectiveness of the entire system.

The ultimate goal is to develop advanced technologies capable of designing and fabricating large-scale structures with atomic-level precision—strategically positioning each atom to create the most effective overall structure for its intended function. While this remains an ambitious objective, following in nature’s footsteps is our most promising route to converging on it.

## 9. Summary and Conclusions

This review has explored nature’s strategies for load-bearing design and highlighted their potential in engineering applications by examining recent research that effectively translates these principles into various technical advancements. The impressive outcomes of these studies underscore the significant benefits of leveraging nature’s strategies to tackle complex engineering challenges. By emulating nature’s holistic approach—where multiple functions are seamlessly integrated into a single structure—engineering design can achieve enhanced performance and versatility. As advancements in computational tools, materials science, and manufacturing techniques continue, the insights gained from studying and replicating nature’s design principles are set to play a crucial role in shaping the next generation of load-bearing engineering solutions.

## Abbreviations

The following abbreviations are used in this manuscript:

AI	Artificial intelligence
CFD	Computational fluid dynamics
CT	Computed tomography
FEM	Finite element method
FGM	Functionally Graded Material
PSL	Principal stress line
SEM	Scanning electron microscopy
SKO	Soft kill option
